# Putative WRKYs associated with regulation of fruit ripening revealed by detailed expression analysis of the WRKY gene family in pepper

**DOI:** 10.1038/srep39000

**Published:** 2016-12-19

**Authors:** Yuan Cheng, Golam JalalAhammed, Jiahong Yu, Zhuping Yao, Meiying Ruan, Qingjing Ye, Zhimiao Li, Rongqing Wang, Kun Feng, Guozhi Zhou, Yuejian Yang, Weiping Diao, Hongjian Wan

**Affiliations:** 1 State Key Laboratory Breeding Base for Zhejiang Sustainable Pest and Disease Control, Institute of Vegetables, Zhejiang Academy of Agricultural Sciences, Hangzhou, China; 2 Department of Horticulture, Zhejiang University, Hangzhou, China; 3 Institute of Vegetables, Jiangsu Academy of Agricultural Sciences, Nanjing, China

## Abstract

WRKY transcription factors play important roles in plant development and stress responses. Here, global expression patterns of pepper *CaWRKY*s in various tissues as well as response to environmental stresses and plant hormones were systematically analyzed, with an emphasis on fruit ripening. The results showed that most *CaWRKY*s were expressed in at least two of the tissues tested. Group I, a subfamily of the entire *CaWRKY* gene family, had a higher expression level in vegetative tissues, whereas groups IIa and III showed relatively lower expression levels. Comparative analysis showed that the constitutively highly expressed *WRKY* genes were conserved in tomato and pepper, suggesting potential functional similarities. Among the identified 61 *CaWRKY*s, almost 60% were expressed during pepper fruit maturation, and the group I genes were in higher proportion during the ripening process, indicating an as-yet unknown function of group I in the fruit maturation process. Further analysis suggested that many *CaWRKY*s expressed during fruit ripening were also regulated by abiotic stresses or plant hormones, indicating that these *CaWRKY*s play roles in the stress-related signaling pathways during fruit ripening. This study provides new insights to the current research on *CaWRKY* and contributes to our knowledge about the global regulatory network in pepper fruit ripening.

Complex and concerted biological processes such as plant growth and development are regulated by various internal and external environmental signals. Transcription factors play an important part in these signaling pathways^1^,^2^. As one of the largest transcription factor families in higher plants, WRKY have been shown to be involved in a multitude of biological processes, including stress response and physiological progress. The common feature of WRKY proteins is the highly conserved N-terminal WRKY domain, which includes an almost conservative peptide (WRKYGQK) and a zinc-finger structure (Cx_4–5_Cx_22–23_HxH or Cx_7_Cx_23_HxC)^3^. The WRKY domain consists of a four-stranded β-sheet and a zinc-binding pocket. It is now known to be the critical structure for DNA binding^4^, which helps to activate or repress the transcription of downstream target regions and regulates the relevant physiological processes.

Since its first identification in sweet potato^5^,^6^, WRKY has been characterized in various plant species and shown to be involved in many biological processes, including biotic and abiotic stress responses^7^,^8^,^9^ and plant–pathogen interactions^9^,^10^. In tomato, the effector-triggered immunity against *Pseudomonas syringae* pv. tomato strain DC3000 (*PstDC3000*) depends on the expression of *SlPR1* and *SlPR1a1* induced by *SlWRKY39*^11^. *SlWRKY12* positively regulates resistance to *Botrytis cinerea*^8^. In pepper, *CaWRKY40* was proved to be involved in the modulation of both high temperature tolerance and *Ralstonia solanacearum* resistance, and this regulation seems to depend on the transcriptional activation of *CaWRKY06*^9^, which is a classical feedback regulation mode of WRKY proteins. In addition, WRKY proteins also play an important role in plant growth and development, such as plant hormone signaling^7^,^12^, secondary signal metabolism^13^,^14^, trichome and seed development^15^,^16^, germination^17^ and leaf senescence^18^,^19^.

As a highly conserved protein family reflected by both conservative WRKY-domain and specific W-box (TTGACC/T) recognition, the diverse regulating function of WRKY is partly due to its interaction with other proteins that forms a complex signal transduction pathway^20^. In *Arabidopsis*, AtWRKY25 and AtWRKY33 interact with AtMKS1 (substrate of AtMPK4) to form a complex, which is subsequently involved in the plant defense response^21^,^22^. Known to regulate endosperm growth and seed size, AtIKU1 also interacts with AtWRKY10^23^. Recent studies showed that a newly identified VQ-motif protein family is one of the most important WRKY interacting proteins, some WRKY and VQ proteins sometimes work together to regulate biological processes^20^. At present, the majority of identified WRKYs are from model plants, such as *Arabidopsis* and rice^24^,^25^.

Klee and Giovannoni^26^ classified fleshy fruits into two physiological types—climacteric (tomato and banana) and non-climacteric (pepper and strawberry). Thus, pepper and tomato present suitable models for highlighting the different types of fruit ripening processes. In the past, little focus was placed on the fruit maturing process of pepper, a non-climacteric fruit, as compared to the climacteric fruit tomato. As a critical stage for pepper yield and quality (size, weight, color, nutrient), fruit ripening can also be affected by many internal and external factors such as hormone signals and biotic or abiotic stresses^11^,^27^,^28^. Transcription factors are also important for fruit ripening^29^,^30^. Recently, different types of transcription factors, including WRKY, MYB, and AP2/EREBP, have been induced during the ripening of pepper fruits^31^. Several *CaWRKY*s have also been reported to be involved in stress responses in pepper^9^,^10^,^32^. However, there are a very few studies on the potential roles of CaWRKY proteins in fruit maturation. The recent elucidation of the whole pepper genome sequence^33^,^34^ (http://peppersequence.genomics.cn/;http://pgd.pepper.snu.ac.kr) will be an asset in deciphering expression patterns and potential functions of all CaWRKY transcription factors during the process of pepper fruit maturation.

Although a comprehensive study of CaWRKYs in pepper was recently performed^35^, little focus was placed on comprehensive expression profiles of the entire WRKY gene family in tissues and their response to abiotic stresses. The global analysis of the *CaWRKY* gene expression pattern will facilitate the functional studies of pepper *CaWRKY*s. We identified 61 *CaWRKY*s and analyzed the expression profiles of all the members in various tissues using RNA-seq and quantitative real-time PCR (qPCR), with emphasis on fruit maturation. Furthermore, comprehensive analysis of the expression patterns of the whole *CaWRKY* gene family under different stress conditions (high salinity, drought, and heat) and in the presence of phytohormones (salicylic acid [SA], jasmonic acid [JA], abscisic acid [ABA] and brassinolide [BR]) revealed the critical and complex regulating roles of CaWRKYs in pepper fruit maturation at the transcriptional level.

## Results

### Classification and expression profile analysis of pepper *CaWRKY* genes

**Figure 1:**
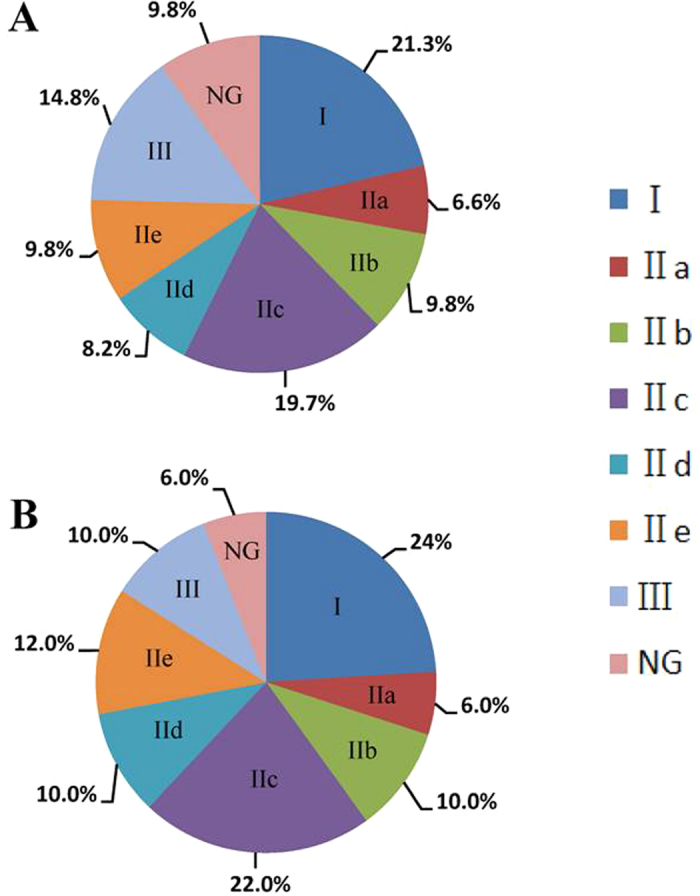
Subfamily constitution of pepper CaWRKYs. (**A**) Group constitution of the identified pepper CaWRKYs. (**B**) CaWRKY genes expressed in the investigated plant tissues. A signal intensity level of over 5.0 is considered to be expressed in the given tissue. 50 of the 61 CaWRKYs are expressed in at least one tested tissue. Percentages of CaWRKY groups are demonstrated in the figure.

A total of 61 potential *CaWRKY*s were identified in the pepper genome (Supplemental Table S1). Based on the number and structure of WRKY domains, 55 of the CaWRKY proteins were classified into three main groups: group I, group II (IIa, IIb, IIc, IId, and IIe), and group III (Supplemental Figure S1; Supplemental Table S2). Two of the CaWRKYs (CaWRKY01 and CaWRKY12) were excluded from phylogenetic analysis as their WRKY domains were incomplete (Supplemental Table S3). The remaining four CaWRKYs (CaWRKY17, CaWRKY19, CaWRKY46, and CaWRKY52) showed evolutionary uniqueness (Supplemental Figure S1) and were not assigned to any group. Thus, all these genes were defined as None-group (NG) in the study. As shown in Fig. 1A, the group II (including IIa, IIb, IIc, IId, and IIe) members had a higher proportion of proteins (54.1%) than did the other groups (I, III and NG). Of the five subgroups within group II, group IIc had the highest proportion of proteins (19.7%), whereas group IIa had the lowest (6.6%).

Using the RNA-seq data of different pepper tissues (root, stem, leaf, bud, flower, and fruit) published previously^33^, we found that 50 *CaWRKY* genes were expressed in at least one tissue (reads per kilobase exon model per million mapped reads [RPKM] ≥5.0 was defined as expressed) (Supplemental Table S4). The highest expression level (RPKM = 487.77) was detected for *CaWRKY28* in leaf. As shown in Fig. 1B, the group II members comprised up to 60% and the group I members 24% of all the 50 expressed *CaWRKY*s. Comparative analysis found that the percentage of WRKYs expressed in group I and group II was higher than that in the remaining two groups (Fig. 1A,B), indicating their higher activity. Among the remaining non-expressed *CaWRKY* genes, 7 out of 11 were derived from group III (*CaWRKY05, CaWRKY29, CaWRKY32* and *CaWRKY49*) and NG (*CaWRKY17, CaWRKY19* and *CaWRKY46*) (Supplemental Tables S2 and S4). Meanwhile, all members of group IId and group IIe *CaWRKY*s were expressed in at least one of the tissues examined (Supplemental Tables S2 and S4).

### *CaWRKY* gene expression in different tissues

**Figure 2:**
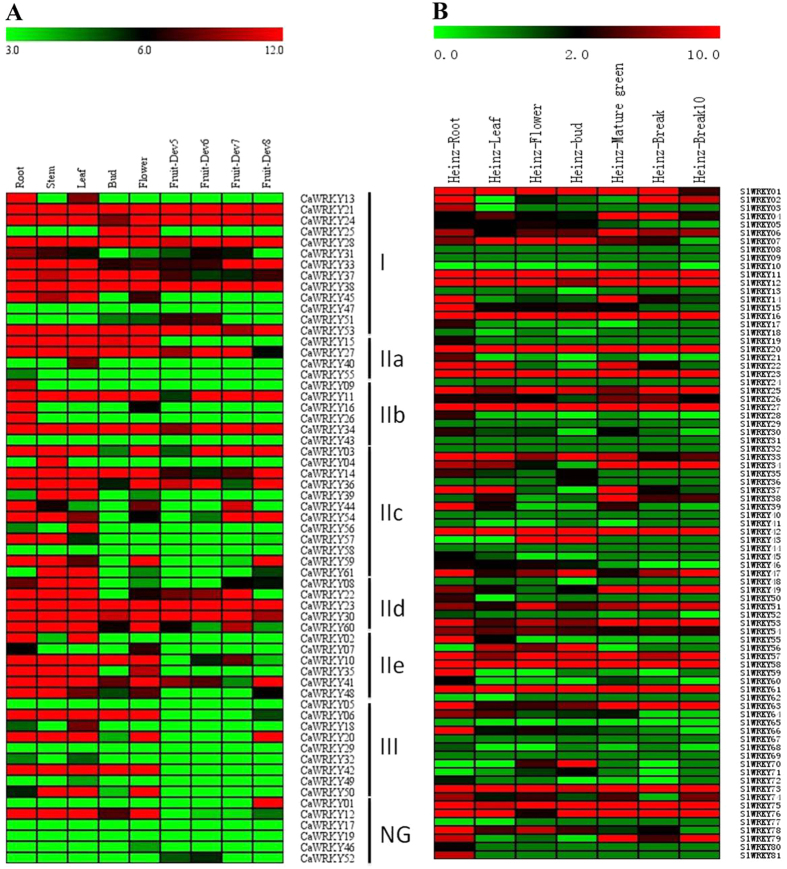
Cluster analysis of tissue-specific expression profiles of pepper (**A**) and tomato (**B**) *WRKY* genes. WRKY gene expression was obtained from the RNA-seq data. WRKY genes and various tissues are represented as rows and columns. Green, black, and red elements indicate low, regular, and high signal intensity, respectively. Cluster analysis was performed based on expression profiles of 61 *CaWRKY*s (**A**) and 81 *SlWRKY*s (**B**) in various tissues (root, stem, leaf, flower, and fruit). CaWRKYs that belong to the same group are clustered together for a more intuitive analysis.

To compare in detail the *CaWRKY* expression in different tissues, the RNA-seq data of the 61 *CaWRKY* genes were selected for further analysis^33^. *CaWRKY* genes with RPKM value ≥5.0 were defined as expressed genes in the specific tissue. The genes expressed only in one tissue were considered to be specifically expressed *CaWRKY*s, and those with the highest expression in specific tissue were regarded as preferentially expressed. According to Table 1, the number of both expressed and preferentially expressed *CaWRKY*s in vegetative tissue (root, stem, and leaf) was higher than that in reproductive tissue (bud, flower, and fruit). Only one or two specifically expressed *CaWRKY* genes were identified in each tested tissue. As shown in Fig. 2A and supplemental Table S4, the expression of some *CaWRKY* genes was tissue specific, such as that of *CaWRKY09* (RPKM = 11.21) and *CaWRKY26* (RPKM = 20.26) that were only expressed in the root, *CaWRKY04* (RPKM = 20.71) that was expressed in the stem, *CaWRKY40* (RPKM = 8.82) and *CaWRKY56* (RPKM = 17.18) that were expressed in the leaf, *CaWRKY01* (RPKM = 31.42) that was expressed in the fully ripened fruit, and *CaWRKY25* (RPKM = 480.15) that was expressed in floral parts. However, most *CaWRKY*s were expressed in two or more tissues. Furthermore, compared to reproductive organs (flower and fruit), the number of preferentially expressed *CaWRKY*s was higher in vegetative parts.

**Figure 3:**
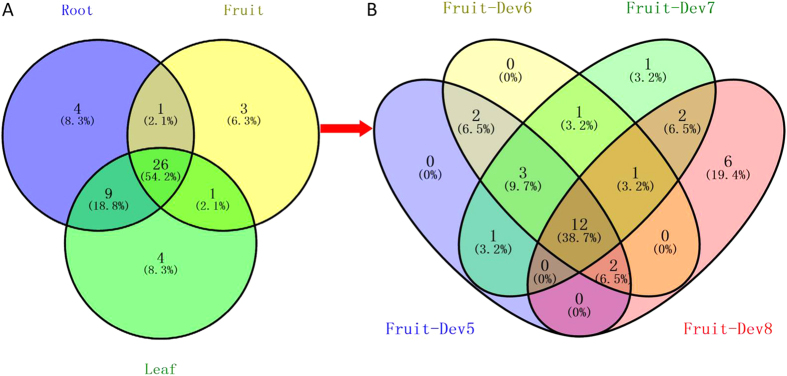
Number of expressed *CaWRKY*s in different tissues. The interaction of vegetative tissues (roots and leaves) and reproductive tissue (fruits) (**A**) and the number of expressed *CaWRKY*s in different fruit developmental stages (Dev5, Dev6, Dev7, Dev8) (**B**).

Based on the *CaWRKY* expression profiles in various tissues (root, leaf, fruit) and different stages of fruit development (Fig. 3), we found that more than half (54.2%) of *CaWRKY*s were simultaneously expressed in the root, leaf, and fruit. Moreover, root and leaf possess many common *CaWRKY*s (Fig. 3A). Further analysis suggested that nearly 40% of *CaWRKY*s were always expressed during the process of fruit ripening (Dev5 to Dev8), and 19.4% of *CaWRKY*s seem to be expressed only at the late stage of fruit maturation (Dev8) (Fig. 3B).

Further analysis of the expression profiles in various tissues revealed that, although group I members possess only 24% of the detected expressed *CaWRKY*s (Fig. 1), the percentage of group I members in reproductive tissues (bud, flower and fruit) and especially in fruits at the early mature stages, was significantly higher (~42%) (Table 2), suggesting the potentially important role of group I *CaWRKY*s during fruit ripening. The subgroup IId presented a similar situation (Table 2). In contrast, although group III contained 10.0% of all expressed *CaWRKY*s, the members of this group were barely detected in fruits. The expression profiles of other groups and subgroups varied among different tissues. For example, the proportion of expressed *CaWRKY*s from subgroups IIa and IIb were well distributed in all the tissues tested, and group IIc had the lowest percentage in the bud (Table 2).

**Figure 4:**
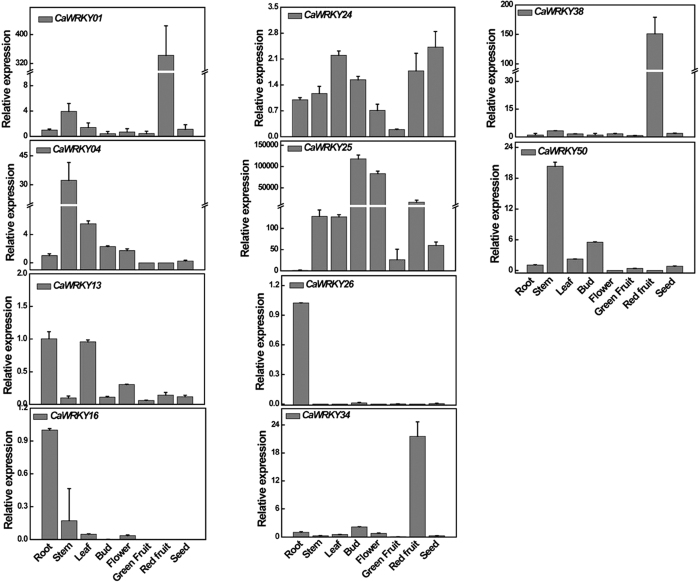
Expression of 12 *CaWRKY* genes in various tissues. The expression levels of these *CaWRKY*s in various tissues (root, stem, leaf, bud, flower, green fruit, red fruit, and seed) were tested using qPCR and the results showed high consistence with the RNA-seq data.

In order to confirm the reliability of RNA-seq data, qPCR was used to test and verify the expression of *CaWRKY* genes in nine specific tissues (root, stem, leaf, bud, flower, green fruit, red fruit, and seed) of the pepper commercial F1 hybrid Zhejiao-3. Ten *CaWRKY* genes with tissue-specific expression patterns were selected for comparison. The results obtained were consistent with the RNA-seq data (Figs 2 and 4), proving the reliability of the RNA-seq data^33^ (http://peppersequence.genomics.cn).

### Conserved constitutive highly expressed *CaWRKY*s in pepper and tomato

Of all the expressed *CaWRKY*s, 16 were highly expressed and found abundantly in each tissue. These were identified (Supplemental Table S4) as group I (5), group II (10) and NG (1) and defined as conserved, constitutively highly expressed *CaWRKY*s (Table 3). The expression levels of these *CaWRKY* genes in most tested tissues were significantly higher than the basic RPKM value (5.0), some of which reaching up to 500 (*CaWRKY28* in leaf, RPKM = 487.77). With the average expression level of 185.97 among all the tested tissues, *CaWRKY28*, a member of group I, had the highest expression in almost every tissue (Supplemental Table S4).

**Figure 5:**
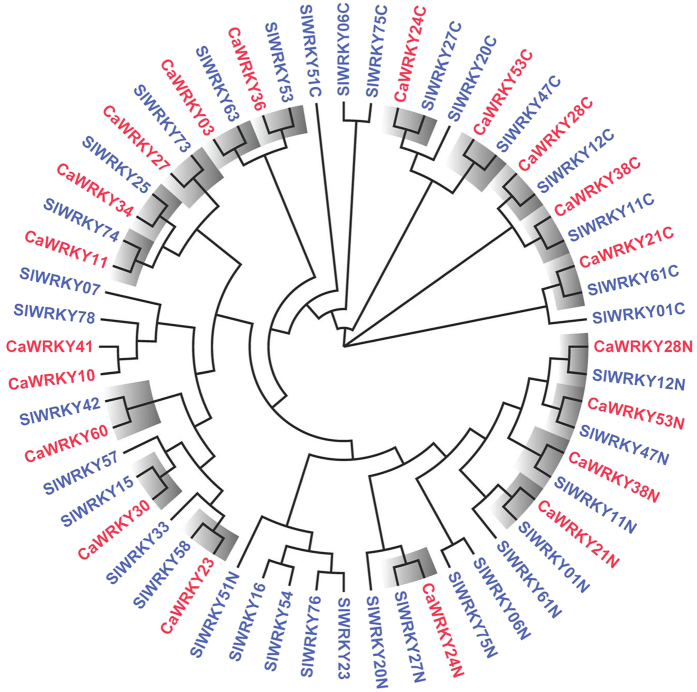
Phylogenetic analysis of constitutively highly expressed *WRKY*s in tomato and pepper. The orthologous genes between tomato and pepper are highlighted in gray.

Additionally, 26 out of 81 constitutively highly expressed tomato WRKYs (SlWRKYs) from various tissues (root, leaf, bud, flower and fruit) were identified (Fig. 2B; Table 3; Supplemental Table S4). Phylogenetic analyses of 16 and 26 SlWRKYs constitutively highly expressed in pepper and tomato, respectively, revealed 14 pairs of orthologous genes (Fig. 5), implying their potential functional conservation. The results showed that, in addition to the resemblance in group constitution between pepper and tomato (Table 3), some SlWRKY members were also evolutionarily conservative with genes of pepper. For example, pepper *CaWRKY28* and tomato *SlWRKY12* were both constitutively highly expressed in various tissues as orthologous proteins. The similar phenomenon was also observed in many other conserved highly expressed WRKY pairs (Fig. 5; Table 3), indicating that the basic expression pattern of WRKY transcription factors might have been formed before the differentiation of *Solanaceae* plants and that they performed conservative and basic functions in plant growth and development.

### Preferentially and specifically expressed *CaWRKY* genes during pepper fruit ripening

According to the expression standard (RPKM ≥5.0), 31 *CaWRKY* genes were expressed during fruit maturation and the highest expression level (RPKM = 200.28) was detected for CaWRKY28 in the late maturation stage of the fruit (Dev8: 5th day after breaker). Among the 31 *CaWRKY* genes, four of them were preferentially expressed at Dev6 (breaker), Dev7 (3rd day after breaker) and Dev8, while only one of the specifically expressed *CaWRKY* was detected in the late maturation stage of the fruit (Dev8) (Table 1; Supplemental Table S4). The five preferentially or specifically expressed *CaWRKY*s belong to group I (*CaWRKY24* in Fruit-Dev7, RPKM = 58.45; *CaWRKY51* in Fruit-Dev6, RPKM = 7.82), group IIb (*CaWRKY11* in Fruit-Dev8, RPKM = 171.69), and NG (*CaWRKY01* in Fruit-Dev8, RPKM = 31.42; *CaWRKY52* in Fruit-Dev6, RPKM = 5.57) (Table 1; Supplemental TableS4). The analysis of the average expression level of *CaWRKY*s suggested that the 31 *CaWRKY*s expressed in fruit are globally induced during the fruit ripening process. The average expression level of group IIb was dramatically increased from 7.24 (Dev5: mature green stage) to 101.70 (Dev8) (Supplemental Table S5). Unlike the other groups, which demonstrated sustained increase in expression during the ripening process, the expression levels of group IIa and group IId attained the highest levels at Dev7 and showed a sudden decrease at Dev8 (Supplemental Table S5).

### *CaWRKY*s expressed during pepper fruit maturation are regulated by different types of stresses

The expression of *CaWRKY* genes in pepper fruit under abiotic stress conditions (high temperature, high salinity, and drought) was analyzed using qPCR. Stress-induced genes were identified (change fold ≤0.5 as down-regulated or change fold ≥1.5 as up-regulated). The results showed that of all *CaWRKY* genes expressed in fruit, 26 and 27 genes were regulated by high temperature and high salinity, respectively, whereas only 14 responded to drought stress (Supplemental Tables S6 and S7). All of the 22 *CaWRKY*s expressed in premature fruits (Dev5 and Dev6), with the exception of *CaWRKY31* and *CaWRKY60*, were regulated by at least one of the tested stress treatments. Meanwhile, all the 30 *CaWRKY*s expressed during the late-maturity period (Dev7 and Dev8), except for *CaWRKY31* and *CaWRKY60*, were regulated by at least one type of stress (Supplemental Tables S6 and S7). As shown in Supplemental Table S6, a much greater number of genes were regulated by high temperature and high salinity than by drought, and most of them were up-regulated. However, when exposed to drought, most *CaWRKY*s seem to be down-regulated (Supplemental Table S6). The high consistency of the regulatory pattern of *CaWRKY* genes under heat and high salinity indicated a close relationship between the two pathways and the function of CaWRKYs in the signaling pathway.

The stress-regulated *CaWRKY* genes demonstrated diverse expression patterns at different maturing stages of pepper fruit. Some stress-induced *CaWRKY*s were constitutively expressed throughout the ripening period, whereas others exhibited different expression patterns during pre-maturation and full maturation stages (Supplemental Table S4). In the late maturity stage, not only were more *CaWRKY*s detected in fruit, but also the expression levels of the corresponding genes increased (Supplemental Table S4), which means that heat-, drought-, and salinity-regulated *CaWRKY* genes preferentially participated in the process at the late maturity stage. The involvement of many heat-, high salinity-, and drought-regulated *CaWRKY*s during fruit maturation indicates an important role of these genes in the response of fruit to abiotic stresses.

### *CaWRKY* expression under hormone treatment

Many *CaWRKY*s have been reported to take part in biological processes regulated by plant hormones^7^,^12^. In our study, the qPCR analysis showed that most *CaWRKY*s expressed in fruit could be regulated by the four hormones tested, namely SA, JA, ABA, and BR (Supplemental Tables S7 and S8). Of all these *CaWRKY* genes, the expression of 24, 25, 24 and 18 genes was significantly changed (induced or repressed) under SA, JA, ABA and BR treatment, respectively (Supplemental Table S8). The number of up-regulated *CaWRKY*s was significantly higher than that of down-regulated genes under SA, ABA, and BR treatments, whereas the number of JA-induced and repressed genes remained almost the same (Supplemental Tables S7 and S8). Furthermore, compared to the other plant hormones, *CaWRKY*s expressed in fruits seemed to be less sensitive to BR. Overall, the expression of *CaWRKY* genes in different stages of fruit maturity demonstrated divergent response profiles under each of the four hormone applications.

The role of ABA in various biological processes, including fruit ripening, seed dormancy and environmental stress responses, has been widely reported^28^,^36^,^37^. However, to better understand the roles of CaWRKYs in the ABA-dependent fruit ripening and stress responses, we further analyzed the *CaWRKY* expression profiles under ABA treatment. The results showed that most of the 31 *CaWRKY*s expressed in fruit were significantly regulated by ABA; 17 were up-regulated and 6 were down-regulated (Supplemental Table S8).

As ABA takes part in various stress responses including those to high salinity, drought, and extreme temperatures^37^, the expression patterns of all the *CaWRKY*s expressed in fruits under ABA were compared under the three abiotic stress (high salinity, drought, and heat) treatments. Almost all ABA-induced *CaWRKY* genes were regulated by at least one of the stresses applied (Supplemental Table S9). These ABA- and stress-regulated *CaWRKY* genes demonstrated a similar trend, especially under ABA, high-salinity, and heat treatments. Most of them were induced, suggesting a common and positive or sometimes redundant role in the ABA and environmental stress signaling pathways.

## Discussion

The WRKY transcription factors, a large protein superfamily, is found abundantly in plants, with hundreds of WRKYs identified up to now^24^,^25^,^38^. The number of WRKY genes in plants range from one in green algae to 75 WRKYs in *Arabidopsis*^3^,^39^. Furthermore, most of these proteins are reported to be involved in defense and resistance processes^40^,^41^,^42^. Recently, the genome of the tomato plant, a representative of the *Solanaceae*, has been found to encode at least 81 SlWRKYs^38^. In our study, 61 CaWRKYs were identified using bioinformatics in pepper. It is well known that tomato and pepper, which represent a typical climacteric and non-climacteric fruit, respectively, have emerged as models for fruit ripening. Our study showed that about 36% of CaWRKYs and 35% of SlWRKYs were expressed in pepper and tomato fruits, respectively (Fig. 2), indicating the potentially critical role of WRKYs in plant growth and development, especially in the regulation of fruit ripening in the two species.

In the present study, 16 *CaWRKY*s were constitutively highly expressed in almost every tissue tested, suggesting their importance and fundamental functions in pepper plant development (Table 3; Fig. 2). At least 25 *SlWRKY*s were also found to be constitutively highly expressed in most tomato tissues. The subfamily constitution of these CaWRKYs and SlWRKYs were found to be very similar (Table 3). Moreover, 13 pairs of orthologous WRKY were identified (Fig. 5), further indicating the potentially conserved role of these WRKYs in plant development in *Solanaceae* plants.

Additionally, the work on *SlWRKY* genes by Huang *et al*.^38^ and qPCR results of pepper *CaWRKY* under biotic and abiotic stress conditions (Supplemental Table S7) indicate that the expression of several pairs of constitutively expressed orthologous *WRKY*s is highly consistent. For example, *CaWRKY36* and *SlWRKY53* are both significantly induced by high salinity and *B. cinerea* infection, *CaWRKY11* and *SlWRKY74* are up-regulated under drought condition. The highly similar stress response pattern of these orthologous *CaWRKY* and *SlWRKY* strongly suggests that the potential roles of WRKY transcription factors in plants are conserved and already formed before the differentiation of tomato and pepper as separate species in the family of *Solanaceae*.

Fruit ripening is a complex biological process and comprises a series of morphological, physiological, and biochemical processes that encompass pigmentation, nutrition, and metabolism of other flavor substances^31^,^43^,^44^. Numerous transcription factors have been reported to take part in the regulation of fruit ripening. For instance, a MADS-box transcription factor gene (*LeMADS-RIN*) defined as a master regulator of ripening, was found to affect all known ripening pathways^29^,^45^. In addition, transcription factors, such as bHLH, NAC, bZIP, and WRKY, also function during tomato or pepper fruit ripening^31^,^46^. In pepper, more than half of the *CaWRKY* genes were expressed at different stages of ripening. Among them, six highly expressed *CaWRKY*s that belonged to either group I (4) or group IId (2) were detected at the early maturation stages (Dev5 and Dev6). In the late stages of pepper maturation (Dev7 and Dev8), not only was the average expression level higher compared to that in the pre-maturity stage (Supplemental Table S5), but also the CaWRKY constitution of groups was more diverse. The diverse constitution of CaWRKYs at this stage reflected the more complicated biological processes at work in this stage of fruit ripening. The significant transcript level changes of numerous *CaWRKY* genes in our study led us to believe that, similar to other maturation-regulating transcription factors identified in tomato (SlNAC1, RIN, and ERF)^44^,^47^,^48^, CaWRKY also plays an important role in the fruit ripening regulatory network.

Early evolutionary origin of the group I WRKYs is supported by the fact that all WRKYs identified in lower plants belong to this group^49^,^50^. We found that group I comprised nearly 25% of the total identified CaWRKYs. Of these, only two CaWRKY members have been functionally identified in disease resistance process (CaWRKY13/Ca02g003339 and CaWRKY45/Ca09g001251)^51^,^52^. Although current studies attribute group I CaWRKYs to disease resistance, our results suggest that group I CaWRKYs play a more important and fundamental role than other groups in reproductive development, especially in ripening of pepper fruit. Group I proteins composed around 24% of all expressed *CaWRKY*s in the five tissues analyzed (root, stem, leaf, bud, flower, and fruit). Interesting, nearly 30% of the expressed *CaWRKY*s during fruit ripening and two of the four *CaWRKYs (CaWRKY24* and *CaWRKY51*) preferentially expressed in fruits belonged to group I. Moreover, the average expression level of group I *CaWRKY* genes was also higher in pepper fruit tissues, and transcription abundance increased along with the ripening process (Supplemental Table S5). In contrast, the remaining group III CaWRKYs were absent during maturation, revealed by their very low expression level in ripening fruits (Table 2; Supplemental Table S5). These results suggest the possible major role of group I CaWRKYs in pepper fruit development, especially in the fruit maturation process.

As a complex and genetically programmed process characterized by changes in the color, texture, flavor, and aroma^30^, fruit maturation is affected by various internal and environmental factors, including different hormones like ethylene (ET), auxin (IAA), and ABA^28^,^31^,^44^ and stress conditions such as salinity, high temperature, or cold^27^,^48^. For example, IAA is known to retard tomato ripening by regulating ET and ABA^28^. The down-regulation of auxin-responsive transcription factor gene (*ARF*) during pepper maturation also indicates the negative role of auxin^31^. Moreover, salinity positively affects the contents of antioxidant compounds at different ripening stages of pepper fruits^27^.

In our study, most *CaWRKY*s expressed in the fruit are regulated by high salinity, drought, or heat and exhibit a similar trend, especially when induced by both high salinity and heat acting together (Supplemental Tables S6 and S7). This phenomenon supported the argument that some abiotic stresses share common gene regulating responses and signal transduction pathways^53^. Among the three abiotic stresses applied, the effect of both high salinity and heat was more significant as compared to drought on *CaWRKY* genes expressed in fruits, indicating that the fruit ripening process might be more sensitive to salinity and high temperature.

As a stress responsive plant hormone, ABA can effectively increase the pigmentation and accelerate ripening of sweet cherry fruits^54^. We found that some *CaWRKY*s simultaneously regulated by abiotic stresses and ABA followed the same pattern, suggesting that these *CaWRKY* genes were regulated by stress-induced ABA in fruit. However, some other *CaWRKY*s demonstrated a different stress-regulated pattern as compared to ABA application. For example, ABA-induced *CaWRKY12* and *CaWRKY37* were both repressed under high temperature, and the expression of drought-repressed *CaWRKY06* increased after ABA treatment (Supplemental Table S7). Comprehensive analysis of CaWRKY expression pattern under ABA treatment revealed that ABA is not only involved in many stress responses, but also reminds us of the complexity of the pepper fruit ripening process, which involves a myriad of CaWRKY transcription factors, interconnected with plant hormones and stress responding pathways.

## Materials and Methods

### Identification of *CaWRKY* encoding genes

The amino acid sequences of *Capsicum annuum* L. ‘Zunla-1’ were downloaded from the pepper genome database (PGD, http://peppersequence.genomics.cn)^33^ and used to construct a local database using the software Bioedit 7.0 (www.softpedia.com/get/Science-CAD/BioEdit.shtml). A Blastp search was performed using the amino acid sequences of the WRKY transcription factors from tomato and potato^38^,^55^. Additionally, the hidden Markov model (HMM) profile of the WRKY domain (Pfam: PF03106) from Pfam 27.0 database (http://pfam.xfam.org/) was used to identify candidate WRKY sequences. The e-value used was 1e^−5^. After excluding the redundant sequences, the remaining sequences were determined by Pfam 27.0.

### Expression analysis of pepper and tomato *WRKY*s in various tissues

Two Illumina RNA-seq datasets downloaded from the PGD and Tomato Functional Genome Database (TFGD, http://ted.bti.cornell.edu/) were used to analyze the expression patterns of WRKY genes in the root, stem, leaf, bud, flower, and fruit tissues of pepper and in the root, leaf, bud, flower, and fruit tissues of tomato, respectively. Fragments per kilobase of exon model per million mapped (FPKM) values were log_2_-transformed and heat maps with hierarchical clustering were exhibited using the software Mev 4.9.0^56^.

### Phylogenetic analysis of *WRKY*s

Amino acid sequences of all WRKYs from pepper and tomato were detected using SMART (http://smart.emblheidelberg.de/). Conserved WRKY domains from WRKY transcription factors in pepper and tomato were aligned using ClustalX version 1.83 with default settings (http://www.clustal.org/). A phylogenetic tree was constructed using the software MEGA 5.05^57^ based on conserved WRKY domains. In the phylogenetic tree, relative branch support was assessed using bootstraps (1000 replicates). Branch lengths were calculated by pairwise comparison of the genetic distances, and missing data were treated by pairwise deletions of gaps.

### Plant materials and stress and hormone treatments

Seeds of the pepper cultivar Zhejiao-3 were germinated in plastic pots with a 3:1 (v/v) mixture of peat and vermiculite and grown in a growth room (25 °C/22 °C, 12 h light/12 h dark). The roots, stems, leaves, buds, and flowers were harvested from 8-week-old pepper plants. The 4-week-old seedlings were sprayed with 10 mM salicylic acid (SA), 100 μM jasmonic acid (JA), 50 μM abscisic acid (ABA), and 10 μM brassinolide (BR) solutions, and leaves were collected 12 h later. For abiotic stress treatments, 4-week-old seedlings were exposed to high salt by culturing them in 150 mM NaCl solution for 12 h, exposed to drought by withholding water for 9 d, or to heat by elevating the temperature to 42 °C for 3 h, and leaves were collected at the end of each treatment. All materials were frozen in liquid nitrogen and used for the following expression analyses.

### RNA isolation, cDNA synthesis, and quantitative RT-PCR analysis

Total RNA from all samples was isolated using TRIZOL reagent and single-stranded cDNA was synthesized following the manufacturer’s procedure (TIANGEN, Beijing, China). Gene-specific primer pairs were designed using GeneScript real-time PCR primer design (www.genescript.com; Supplemental Table S10) and further verified for their specificity by observing a single amplicon during electrophoresis. qPCR reactions were carried out using the following program: 10 min at 94 °C, 30 cycles of 45 s at 94 °C, 45 s at 55 °C, and 1 min at 72 °C, followed by an extension of 7 min at 72 °C. The ubiquitin-conjugating enzyme genes (UBI1-F:AAGGAAATGTGTGTCTCAAC; UBI1-R: TCCAAATGCCAAACTTCTAG) were used as a reference. Relative gene expression was calculated in accordance with the methods described by Livak and Schmittgen^58^.

## Additional Information

**How to cite this article**: Cheng, Y. *et al*. Putative WRKYs associated with regulation of fruit ripening revealed by detailed expression analysis of the WRKY gene family in pepper. *Sci. Rep.* **6**, 39000; doi: 10.1038/srep39000 (2016).

**Publisher's note:** Springer Nature remains neutral with regard to jurisdictional claims in published maps and institutional affiliations.

## Supplementary Material

Supplementary Table

## Figures and Tables

**Table 1:** The number of expressed, preferentially expressed, and specifically expressed *CaWRKY* genes in different tissues.

**Type**	Root	Stem	Leaf	Bud	Flower	Fruit-Dev5	Fruit-Dev6	Fruit-Dev7	Fruit-Dev8
Expressed *CaWRKY*s	40	37	40	23	34	20	21	21	23
Preferentially expressed *CaWRKY*s	15	7	17	1	4	0	2	1	1
Specifically expressed *CaWRKY*s	2	1	2	0	0	0	0	0	1

**Table 2:** Subfamily constitution of expressed *CaWRKY* genes in different tissues of pepper plants.

Type	Root	%	Stem	%	Leaf	%	Bud	%	Flower	%	Fruit								Total	%
											Dev5	%	Dev6	%	Dev7	%	Dev8	%		
I	10	25.6	9	24.3	10	24.4	8	33.3	9	26.5	9	45.0	9	42.9	8	38.1	7	29.2	12	23.5
IIa	2	5.1	2	5.5	3	7.3	2	8.3	2	5.9	1	5.0	1	4.8	1	4.8	1	4.2	3	5.9
IIb	5	12.8	2	5.4	2	4.9	2	8.3	3	8.8	2	10.0	2	9.5	2	9.5	2	8.3	5	9.8
IIc	7	17.9	10	27.0	9	22.0	2	8.3	6	17.6	2	10.0	3	14.3	4	19.5	6	25.0	11	21.6
IId	5	12.8	5	13.5	5	12.2	3	12.5	4	11.8	4	20.0	3	14.3	5	23.8	3	12.5	5	9.8
IIe	5	12.8	4	10.8	5	12.2	4	16.7	5	14.7	1	5.0	2	9.5	1	4.8	2	8.3	6	11.8
III	4	10.3	4	10.8	6	14.6	2	8.3	4	11.8	0	0.0	0	0.0	0	0.0	2	8.3	6	11.8
NG	1	2.6	1	2.7	1	2.4	1	4.2	1	2.9	1	5.0	1	4.8	0	0.0	1	4.2	3	5.9
Total	39	100	37	100	41	100	24	100	34	100	20	100	21	100	21	100	24	100	54	100

NG: None-group, genes that were not assigned to any group; Dev 5: Mature green, the full size green fruit (5–6 cm); Dev6: Breaker, fruit turning red; Dev7: Breaker plus 3days; Dev8: Breaker plus 5 days.

**Table 3:** Constitutively highly expressed CaWRKYs in pepper, tomato, and potato.

Name	Locus no.	Type	Name	Locus no.	Type
CaWRKY28	Capana06g001506	I	SlWRKY11	solyc07g066220.2.1	I
CaWRKY53	Capana11g001882	I	SlWRKY12	solyc06g066370.2.1	I
CaWRKY38	Capana07g002454	I	SlWRKY01	solyc12g014610.1.1	I
CaWRKY21	Capana03g003085	I	SlWRKY61	solyc07g065260.2.1	I
CaWRKY24	Capana04g001820	I	SlWRKY27	solyc03g104810.2.1	I
CaWRKY27	Capana06g001110	IIa	SlWRKY47	solyc05g012770.2.1	I
CaWRKY11	Capana02g002230	IIb	SlWRKY75	solyc12g006170.1.1	I
CaWRKY34	Capana07g001387	IIb	SlWRKY06	solyc07g047960.2.1	I
CaWRKY03	Capana01g002803	IIc	SlWRKY51	solyc07g005650.2.1	I
CaWRKY36	Capana07g001968	IIc	SlWRKY73	solyc06g068460.2.1	IIa
CaWRKY23	Capana04g000568	IId	SlWRKY25	solyc07g051840.2.1	IIb
CaWRKY60	Capana00g003083	IId	SlWRKY74	solyc02g080890.2.1	IIb
CaWRKY30	Capana06g003072	IId	SlWRKY33	solyc02g093050.2.1	IIc
CaWRKY41	Capana08g001012	IIe	SlWRKY20	solyc02g088340.2.1	IIc
CaWRKY10	Capana02g001642	IIe	SlWRKY63	solyc01g079260.2.1	IIc
CaWRKY12	Capana02g003053	NG	SlWRKY53	solyc05g012500.2.1	IIc
			SlWRKY42	solyc08g006320.2.1	IId
			SlWRKY58	solyc04g078550.2.1	IId
			SlWRKY78	solyc07g055280.2.1	IId
			SlWRKY57	solyc06g008610.2.1	IId
			SlWRKY15	solyc09g066010.1.1	IId
			SlWRKY07	solyc01g079360.2.1	IId
			SlWRKY16	solyc01g095630.2.1	III
			SlWRKY76	solyc03g095770.2.1	III
			SlWRKY23	solyc09g015770.2.1	III
			SlWRKY54	solyc05g050330.2.1	III

NG: None-group, genes that were not assigned to any group.
