# Corrigendum: Putative WRKYs associated with regulation of fruit ripening revealed by detailed expression analysis of the WRKY gene family in pepper

**DOI:** 10.1038/srep43498

**Published:** 2017-04-24

**Authors:** Yuan Cheng, Golam Jalal Ahammed, Jiahong Yu, Zhuping Yao, Meiying Ruan, Qingjing Ye, Zhimiao Li, Rongqing Wang, Kun Feng, Guozhi Zhou, Yuejian Yang, Weiping Diao, Hongjian Wan

Scientific Reports 6: Article number: 3900010.1038/srep39000; published online 12 19 2016; updated on 04 24 2017

The original version of this Article contained a typographic error in the spelling of Golam Jalal Ahammed, which was incorrectly given as Golam JalalAhammed, This error has now been fixed in the HTML and PDF versions of this Article.

