# Supplementary material for: Putative WRKYs associated with regulation of fruit ripening revealed by detailed expression analysis of the WRKY gene family in pepper
Source: Sci Rep. 2016 Dec 19;6:39000. doi: 10.1038/srep39000 (PMC5171846; doi:10.1038/srep39000)
Supplement: Supplementary Table [file srep39000-s1.pdf]

Putative WRKYs associated with regulation of fruit ripening revealed by detailed expression analysis of the WRKY gene family in pepper

Yuan Cheng[1], Golam Jalal Ahammed[2], Jiahong Yu[1], Zhuping Yao[1], Meiying Ruan[1], Qingjing Ye[1], Zhimiao Li[1], Rongqing Wang[1], Kun Feng[1], Guozhi Zhou[1], Yuejian Yang[1], Weiping Diao[3], Hongjian Wan[1]*

[1]State key Laboratory Breeding Base for Zhejiang Sustainable Pest and Disease Control, Institute of Vegetables, Zhejiang Academy of Agricultural Sciences, Hangzhou, China

[2]Department of Horticulture, Zhejiang University, Hangzhou, China

[3]Institute of Vegetables, Jiangsu Academy of Agricultural Sciences, Nanjing, China

Tel: +86-571-86404352 Fax: +86-571-86400997

*Corresponding to Dr. Hongjian Wan, E-mail: hjwan@mail.zaas.ac.cn

Supplemental Table S1 The WRKY gene family in pepper

Name	Locus	Chromosome
CaWRKY01	Capana01g000165	[mRNA] locus=Chr01:2380410:2384292
CaWRKY02	Capana01g000167	[mRNA] locus=Chr01:2417686:2419547
CaWRKY03	Capana01g002803	[mRNA] locus=Chr01:178651461:178653597
CaWRKY04	Capana01g003441	[mRNA] locus=Chr01:226400966:226405165
CaWRKY05	Capana01g004471	[mRNA] locus=Chr01:300738347:300739954
CaWRKY06	Capana01g004472	[mRNA] locus=Chr01:300742280:300745209
CaWRKY07	Capana02g000212	[mRNA] locus=Chr02:26415840:26420359
CaWRKY08	Capana02g000680	[mRNA] locus=Chr02:83748217:83749821
CaWRKY09	Capana02g000918	[mRNA] locus=Chr02:102411100:102416945
CaWRKY10	Capana02g001642	[mRNA] locus=Chr02:129707611:129709054
CaWRKY11	Capana02g002230	[mRNA] locus=Chr02:142213621:142216150
CaWRKY12	Capana02g003053	[mRNA] locus=Chr02:154730960:154732560
CaWRKY13	Capana02g003339	[mRNA] locus=Chr02:159222405:159225272
CaWRKY14	Capana02g003661	[mRNA] locus=Chr02:163357641:163359108
CaWRKY15	Capana03g000473	[mRNA] locus=Chr03:6501568:6503375
CaWRKY16	Capana03g001099	[mRNA] locus=Chr03:18684171:18687268
CaWRKY17	Capana03g001962	[mRNA] locus=Chr03:39904102:39904887
CaWRKY18	Capana03g002072	[mRNA] locus=Chr03:44184049:44185319
CaWRKY19	Capana03g002134	[mRNA] locus=Chr03:48509522:48510754
CaWRKY20	Capana03g002635	[mRNA] locus=Chr03:93204626:93207341
CaWRKY21	Capana03g003085	[mRNA] locus=Chr03:167540037:167548534
CaWRKY22	Capana03g003279	[mRNA] locus=Chr03:203453030:203454904
CaWRKY23	Capana04g000568	[mRNA] locus=Chr04:9062869:9064395
CaWRKY24	Capana04g001820	[mRNA] locus=Chr04:115696954:115705628

CaWRKY25	Capana05g002502	[mRNA]	locus=Chr05:216311579:216315172
CaWRKY26	Capana06g001008	[mRNA]	locus=Chr06:17571223:17578663
CaWRKY27	Capana06g001110	[mRNA]	locus=Chr06:20042156:20043655
CaWRKY28	Capana06g001506	[mRNA]	locus=Chr06:35621649:35624167
CaWRKY29	Capana06g002128	[mRNA]	locus=Chr06:92548747:92550798
CaWRKY30	Capana06g003072	[mRNA]	locus=Chr06:218689176:218691106
CaWRKY31	Capana07g000181	[mRNA]	locus=Chr07:9032674:9041137
CaWRKY32	Capana07g000528	[mRNA]	locus=Chr07:40750931:40752123
CaWRKY33	Capana07g001256	[mRNA]	locus=Chr07:167387734:167411421
CaWRKY34	Capana07g001387	[mRNA]	locus=Chr07:177671908:177674692
CaWRKY35	Capana07g001809	[mRNA]	locus=Chr07:204092824:204096737
CaWRKY36	Capana07g001968	[mRNA]	locus=Chr07:210180549:210184089
CaWRKY37	Capana07g002350	[mRNA]	locus=Chr07:219055519:219061043
CaWRKY38	Capana07g002454	[mRNA]	locus=Chr07:220545442:220548961
CaWRKY39	Capana08g000429	[mRNA]	locus=Chr08:60198759:60200111
CaWRKY40	Capana08g000683	[mRNA]	locus=Chr08:101219429:101222082
CaWRKY41	Capana08g001012	[mRNA]	locus=Chr08:123573760:123575138
CaWRKY42	Capana08g001044	[mRNA]	locus=Chr08:124129873:124131797
CaWRKY43	Capana08g001961	[mRNA]	locus=Chr08:139093292:139095397
CaWRKY44	Capana09g000676	[mRNA]	locus=Chr09:32179774:32181439
CaWRKY45	Capana09g001251	[mRNA]	locus=Chr09:129708552:129712643
CaWRKY46	Capana09g001790	[mRNA]	locus=Chr09:205298779:205300747
CaWRKY47	Capana10g000205	[mRNA]	locus=Chr10:3656310:3659300
CaWRKY48	Capana10g000754	[mRNA]	locus=Chr10:46615318:46617494
CaWRKY49	Capana10g001220	[mRNA]	locus=Chr10:128628965:128629689
CaWRKY50	Capana10g001548	[mRNA]	locus=Chr10:164830295:164833345

CaWRKY51	Capana10g001791	[mRNA]	locus=Chr10:183798726:183801612
CaWRKY52	Capana10g001805	[mRNA]	locus=Chr10:185896489:185897886
CaWRKY53	Capana11g001882	[mRNA]	locus=Chr11:200570029:200575124
CaWRKY54	Capana11g001905	[mRNA]	locus=Chr11:201981442:201987021
CaWRKY55	Capana12g001134	[mRNA]	locus=Chr12:48289811:48291121
CaWRKY56	Capana12g001826	[mRNA]	locus=Chr12:170920299:170933933
CaWRKY57	Capana12g001851	[mRNA]	locus=Chr12:173653320:173658337
CaWRKY58	Capana00g000429	[mRNA]	locus=Chr00:245677269:245678729
CaWRKY59	Capana00g001033	[mRNA]	locus=Chr00:331116291:331117751
CaWRKY60	Capana00g003083	[mRNA]	locus=Chr00:523068129:523069793
CaWRKY61	Capana00g004112	[mRNA]	locus=Chr00:612680042:612682368

Supplemental Table S2. Classification of CaWRKY in pepper based on phylogenetic tree (Supplement Figure S1)

Group Ⅰ	Group Ⅱa	Group Ⅱb	Group Ⅱc	Group Ⅱd	Group Ⅱe	GroupⅢ	None group
CaWRKY13	CaWRKY15	CaWRKY09	CaWRKY03	CaWRKY08	CaWRKY02	CaWRKY05	CaWRKY01
CaWRKY21	CaWRKY27	CaWRKY11	CaWRKY04	CaWRKY22	CaWRKY07	CaWRKY06	CaWRKY12
CaWRKY24	CaWRKY40	CaWRKY16	CaWRKY14	CaWRKY23	CaWRKY10	CaWRKY18	CaWRKY52
CaWRKY25	CaWRKY55	CaWRKY26	CaWRKY36	CaWRKY30	CaWRKY35	CaWRKY20	CaWRKY17
CaWRKY28		CaWRKY34	CaWRKY39	CaWRKY60	CaWRKY41	CaWRKY29	CaWRKY19
CaWRKY31		CaWRKY43	CaWRKY44		CaWRKY48	CaWRKY32	CaWRKY46
CaWRKY33			CaWRKY54			CaWRKY42	
CaWRKY37			CaWRKY56			CaWRKY49	
CaWRKY38			CaWRKY57			CaWRKY50	
CaWRKY45			CaWRKY58				
CaWRKY47			CaWRKY59				
CaWRKY51			CaWRKY61				
CaWRKY53							

Supplemental Table S3. Amino acid sequences of WRKY domain of WRKY genes in pepper

Name	WRKY domain
CaWRKY01	LDDGFKWRKYGQKAVKNSTHPRD (Incomplete)
CaWRKY02	SDKWAWRKYGQKPIKGSPYPRSYYRCSSSkGCLARKQVEQSCTEHGIFIVTYTAEHNHS
CaWRKY03	EDGYRWRKYGQKAVKNSPFPRNYYRCTSATCNVKKRVERCFSDPSIVVTTYEGK
CaWRKY04	LDDGYKWRKYGQKVVKNSLHPRSYYRCTHSNCRVKKRVERLSEDCRMVITTYEGRHNHS
CaWRKY05	DDGYSWRKYGQKHILGAKYPRSYYRCTYRhmqNCWATKQVQRSDDDATVYEITYRGSHNCR
CaWRKY06	DDGYSWRKYGQKDILGAKYPRSYYRCTYRhmqNCWATKQVQRSDDDPTVFDVTYRGSHSCH
CaWRKY07	SDLWAWRKYGQKPIKGSPYPRGYYRCSSSkGCSARKQVERSRTDPNMLVITYTSEHNHP
CaWRKY08	DDYSWRKYGQKPIKGSPHPRAYYKCSSVrGCPARKHVERALDEPTMLVVTYESEHNHS
CaWRKY09	NDGCQWRKYGQKISRGNPCPRAYYRCSVApLCPVRKQVQRCLEDMSILITTYEGTHNHS
CaWRKY10	SDSWAWRKYGQKPIKGSPYPRGYYRCSSSkGCPARKQVERSRADPNMLIVTYSCEHNHP
CaWRKY11	SDGCQWRKYGQKMAKGNPCPRAYYRCTMAvGCPVRKQVQRCAEDRTILITTYEGTHNHP
CaWRKY12	DDYSWRKYGQKPIKGSPHP (Incomplete)
CaWRKY13N	DGYNWRKYGQKKVKATECPRSYYKCTHLKCPAKKKVEKSV-DGHITEITYNGRHNHA
CaWRKY13C	LDDGYRWRKYGQKAVKGTQHPRSYYRCTYAGCNVRKQVERASTDPKAVITTYEGKHNHD
CaWRKY14	LDDGYRWRKYGQKAVKNNNYPRSYYRCTHEGCNVKKQVQRLSKDEGVVVTTYEGMHTHP
CaWRKY15	VKDGYQWRKYGQKVTRDNPSPRAYFKCSFApTCPVKKKVQRSLEDQSILVATYEGEHNHSK
CaWRKY16	MNDGCQWRKYGQKIAKGNPCPRAYYRCTVApSCPVRKQVQRCIQDMSILIITYEGTHNHP
CaWRKY17	ADQWRWRKYGMKRTGGSPFLKSYYRCNQGeDCPARRHVQQSSTDSNKVIVTYRGQHSHP
CaWRKY18	LDDGFSWRKYGQKDILGAKHPRGYYRCTLRhvqGCLATKQVQRSDEDPTIFEVTYRGRHTCS
CaWRKY19	DDIWSWRKYGQKFIKGSSFPRNYFKCNTSeLCQARKQIEKSSKNDCFFLVAYSGMHNHD
CaWRKY20	MNDGCAWRKYGQKSILNSKYPRCYYRCTHKydqDCRATKQVHIMQENPKlMYHTTYFGNHTCN

CaWRKY21N	EDGYNWRKYGQKLVKGSEFPRSYYKCTYPNCEVKKIFERPP-DGQITEIVYKGSHDHPK
CaWRKY21C	LDDGYRWRKYGQKVVRGNPNPRSYYKCTNARCPVRKHVERASHDPKAVITTYEGKHNHD
CaWRKY22	DEYSWRKYGQKPIKGSPHPRGYYKCSSMrGCPARKHVERCLEEPSMLIVTYEGEHNHS
CaWRKY23	DDYSWRKYGQKPIKGSPHPRGYYKCSSVrGCPARKHVERALDDPAMLIVTYEGEHNHS
CaWRKY24N	SDGYNWRKYGQKMVKASECPRSYYKCTHVKCPVRKKVERSV-DGHVTEITYKGHHNHE
CaWRKY24C	LDDGFKWRKYGQKVVKGNHHPRSYYRCTYPGCNVRKHVERASTDPKAVITTYEGKHNHE
CaWRKY25N	DDGYTWRKYGQKHVKGSNFPRSYYKCTQQTCPVRKKVECAP-NGQVIEIVYNGPHNHPK
CaWRKY25C	EDGYRWRKYGQKVVKGNPNPRSYYKCTSAGCLVRKHVERASDDLKSVITTYEGKHNHE
CaWRKY26	MNDGCQWRKYGQKIAKGNPCPRAYYRCTVApNCPVRKQVQRCAEDMSILITTYEGTHNHT
CaWRKY27	VKDGYQWRKYGQKVTRDNPCPRAYFRCSFApTCPVKKKVQRSIEDQSIVVATYEGEHNHP
CaWRKY28N	DDGYNWRKYGQKQVKGSENPRSYYKCTYPNCPTKKKVERSL-DGQITEIVYKGNHNHPK
CaWRKY28C	LDDGYRWRKYGQKVVKGNPNPRSYYKCTSPGCPVRKHVERASQDIKSVITTYEGKHNHD
CaWRKY29	EDGYTWRKYGQKEILGSRFPRAYYRCTHQklyHCPAKKQVQRLDNDPYVFEVTYRSQHIC
CaWRKY30	SDEYSWRKYGQKPIKGSPHPRGYYKCSSMrGCPARKHVERCLEDPSMLIVTYEGEHNHP
CaWRKY31N	RDGYNWRKYGQKQVKSPQGTRSYYRCTHFECCAKK-IECSGHTNRVMEIIYRSEHNHD
CaWRKY31C	SDGYRWRKYGQKMVKGNPHPRNYYRCSSAGCPVRKHIERAVDSTIALTITYKGVHDHD
CaWRKY32	EDGYSWRKYGQKLIVGAKYPREHYRCDCRrlsYREATKMVQRSEAEPLSFEVTYGGSNS
CaWRKY33N	EDGYNWRKYGQKLVRGNMFTRSYYKCTHSNCLAKKQVERSH-DGHITNIQYITNHEHPK
CaWRKY33C	LNDGYRWRKYGQKFVKGNPNPRSYYRCSSAGCPAKKHVERASHDPKLVITTYEGQHEHD
CaWRKY34	TDGCQWRKYGQKMAKGNPCPRAYYRCTMAaGCPVRKQVQRCAEDRTILITTYEGTHNHP
CaWRKY35	DSWSWRKYGQKPIKGSPYPRGYYRCSSSkGCPARKQVERSCLDPTMLLITYCSDHNHQ
CaWRKY36	EDGYRWRKYGQKAVKNSPFPRSYYRCTSQKCSVKKRVERSYQDPSIVITTYEGQHNHH
CaWRKY37N	DDGYNWRKYGQKLVKGSEFPRSYYKCTYPNCEVKKIFERSP-EGQITEIVYKGSHDHPK
CaWRKY37C	LDDGYKWRKYGQKVVRGNPNPRSYYKCTNAGCPVRKHVERASHDPKAVITTYEGKHNHD

CaWRKY38N	EDGYNWRKYGQKQVKGSEYPRSYYKCTHPNCPVKKKVERSQ-EGHITEIIYKGAHNHPK
CaWRKY38C	LDDGYRWRKYGQKVVKGNPNPRSYYKCTSAGCNVRKHVERASHDLKSVITTYEGKHNHD
CaWRKY39	LDDGYKWRKYGKKMVKNSPNPRNYYRCSVEGCPVKKRVERDKEDSRYVITTYEGVHNHQ
CaWRKY40	VKDGYNWRKYGQKVTRDNPYPRAYYKCSFApTCPVKKKVQRSIEDPSILVAVYEGEHNHP
CaWRKY41	SDMWSWRKYGQKPIKGSPYPRGYYRCSTSkGCLARKQVERNRSDPNMFIVTYTAEHNHP
CaWRKY42	EDGFSWRKYGQKDILGANHPRAYYRCTHRhtqGCLATKQVQRSDGNSTIFEVTYKGRHSCK
CaWRKY43	MNDGCQWRKYGQKIAKGSPnCPRAYYRCTVApGCPVRKQVQRCLEDMSILITTYEGTHNHP
CaWRKY44	EDGYRWRKYGQKAVKNSPFPRNYYRCTTQKCSVKKRVERSYEDASIVITTYEGQHNHH
CaWRKY45N	EDGYNWRKYGQKQVKGSENPRSYYKCTFPNCPTKKKVERNL-DGHVTEIVYKGSHNHPK
CaWRKY45C	LDDGYRWRKYGQKVVKGNPNPRSYYKCTFIGCPVRKHVERASHDLRAVITTYEGKHNHD
CaWRKY46	DDGYKWRKYGQKSIKNSPYPRSYYKCTNPRCGAKKQVERSSNEPDTFIITYEGLHLH
CaWRKY47N	EDGYNWRKYGQNQANGRTYPRSYYKCAYPKCPVKKRVGGYH-DCQVMEIIYKGIHNHPK
CaWRKY47C	KRRSYYKCTSSGCNVRKHIQRSPYDQKSVITTYDGKHYHE
CaWRKY48	SDMWSWRKYGQKPIKGSPYPRGYYKCSTSkACLARKQVERNRSDPNMFIVTYTAEHNHP
CaWRKY49	VDDGHVWRKYGQKEILNFPHPRNYYRCTHKfdrGCEATKQVQRIQENPPKFRTTYQGHHSCT
CaWRKY50	VDDGHGWRKYGQKQILNAKFPRNYFRCTHKfdqGCQASKQVQRIQENPPLFRITYYGHHTCK
CaWRKY51N	DGYNWRKYGQKKVKGSEYPRSYYKCTHLKCPVKKKVERSY-DGQITEIVYRGDHNHPK
CaWRKY51C	EDGFRWRKYGHKVVKGSSYPRSYYRCTSPKCSVRKFVERTTDDPRAFITTYEGKHNH
CaWRKY52	DGYNWRKYGQKQVKGSEYPRSYYKCTHLKCLVKKKVERSY-DGQITEIVYRGEHNHPK
CaWRKY53N	DDGYNWRKYGQKQVKGSEYPRSYYKCTQPNCPVKKKVERSL-DGQVTEIIYKGQHNHQ
CaWRKY53C	LDDGYRWRKYGQKVVKGNPYPRSYYKCTSQGCNVRKHVERAASDPKAVITTYEGKHNHD
CaWRKY54	EDGYRWRKYGQKAVKNSPFPRSYYRCTNTKCTVKKRVERSSEDSSIVITTYEGQHCHH
CaWRKY55	VKDGYNWRKYGQKVTRDNPSPRAYYKCSFApTCPVKKKVQRSVKDPSVLVATYEGEHNHP
CaWRKY56	LDDGYKWRKYGKKMVKNSPNPRNYYKCSNGGCNVKKRVERDNEDSSYVITTYEGIHNHE

CaWRKY57	LDDGYKWRKYGQKVVKNTQHPRSYYRCTQDNCRVKKRVERLAEDPRMVITTYEGRHVHS
CaWRKY58	KKKGEKKIKKPRSYYRCTHQGCNVKKQVQRLSKDEGVVVTTYEGMHSHP
CaWRKY59	LDDGYRWRKYGQKAVKNNKFPRSYYRCTHQGCNVKKQVQRLSKDEGVVVTTYEGMHSHP
CaWRKY60	ADEFTWRKYGQKPIKGSPYPRGYYRCSSLkGCPARKHVERATDDPRMLIVTYENDHEHH
CaWRKY61	LDDGYKWRKYGKKKVKSNTNLRNYYKCSSGDCKVKKRVERDGNDSSYLITTYEGRHNHE

Supplemental Table S4. Fragments per kilobase of exon model per million mapped (FPKM) values of all the CaWRKYs in different pepper (Zunla-1)tissues

Name	pepper tissues								
	Root	Stem	Leaf	Bud	Flower	Fruit-Dev5	Fruit-Dev6	Fruit-Dev7	Fruit-Dev8
CaWRKY01	0.4910948	0.1239128	0.2750121	0	0	0	0	0.45114178	31.41895655
CaWRKY02	25.693427	3.7984634	12.726526	0.5705227	2.7314436	0	0	0	0.542759526
CaWRKY03	99.455847	56.36566	55.867869	4.5604284	19.407626	4.990421537	12.70307388	36.09233178	25.06692022
CaWRKY04	1.954148	20.70893	0.8754553	0.3851028	1.6496482	0	0.178784003	0	0.18318134
CaWRKY05	0	0.1908657	0.6354111	0	0.0939079	0	0	0.086862984	0
CaWRKY06	34.251444	86.09697	334.29294	18.648681	54.922053	0.08959182	0.760183949	1.441788741	5.192541924
CaWRKY07	6.0077938	1.695182	2.0981986	0.9548004	7.1214759	0	0	0	0.12111163
CaWRKY08	8.3208882	19.595547	38.548272	0.3726802	4.2571566	0.73407491	1.614823251	6.254134843	5.790893977
CaWRKY09	11.210638	0.7785312	0.230383	0	0	0	0.094096844	0	0
CaWRKY10	68.883716	75.673234	95.464942	16.037506	50.255536	3.069109245	5.645810613	8.361705137	3.760038033
CaWRKY11	73.369374	42.672947	134.40228	26.388297	102.99274	5.387386886	24.82659675	84.86250309	171.6908742
CaWRKY12	109.84369	144.41754	170.80017	7.8287353	55.235125	2.769683953	3.599204266	3.401371581	4.700047542
CaWRKY13	17.623745	1.1760173	8.9720571	0.4305498	0.2893062	0	0.266510315	0	1.092261407
CaWRKY14	17.220929	151.09067	150.72291	19.186374	81.356074	7.263736776	5.555074371	8.078257506	27.133736
CaWRKY15	50.147443	73.821149	133.35536	11.521082	50.578101	0	0.237718342	0.119346371	1.704956517
CaWRKY16	22.872551	1.4564522	3.2324502	1.56411	5.9476909	0	0	0	0.811634245
CaWRKY17	0	0	0	0.0881915	0	0.173712383	0	0	0.419499252
CaWRKY18	4.9334228	1.0993031	8.6110355	0.2525265	0.8272103	0	0	0	0
CaWRKY19	0	0	0	0	0	0.405157072	0	0	0
CaWRKY20	33.455013	39.130017	91.747713	4.0050695	12.886664	0.075854407	3.003571246	1.866975068	30.11501231
CaWRKY21	33.407379	46.260653	49.24968	19.77319	23.100422	33.93163117	32.61576556	23.53212559	16.92274941

CaWRKY22	41.106078	30.280285	17.148624	2.2426577	6.6442578	8.500155648	9.149534258	13.6855186	2.909350695
CaWRKY23	342.32664	278.27678	189.7568	45.027409	135.95291	30.60109425	25.06032175	46.33682368	25.23831797
CaWRKY24	23.545881	37.586466	43.593367	8.9962548	21.046139	18.69935493	24.31169349	58.44597234	34.95460325
CaWRKY25	0	0.0931785	0	426.05413	54.096847	0	0	0	0
CaWRKY26	20.258825	0.0748967	0.3740078	0.0731208	0.2210995	0	0	0	0
CaWRKY27	58.753996	263.02139	228.53736	28.276336	34.033041	9.680866353	29.21784973	52.84340821	5.890140327
CaWRKY28	214.72135	270.98899	487.76732	78.156937	262.41655	11.06728238	39.97727537	108.3771572	200.2782652
CaWRKY29	0.2056998	0.7266291	1.7278722	0	0	0	0	0	0
CaWRKY30	37.589903	22.69229	16.918911	10.730956	16.136678	24.80183432	18.80245242	26.786332	9.797114368
CaWRKY31	9.4621902	7.4219976	5.8171699	3.0402856	4.1879614	5.040325752	6.35153694	5.763435179	2.98874818
CaWRKY32	4.500462	3.1078337	4.9078553	0	0.9409758	0.459723681	0.541769705	0.217596162	0.55509497
CaWRKY33	17.061733	14.445244	13.584651	6.9052923	7.7362812	7.166933661	7.373298872	12.2814275	12.25419999
CaWRKY34	29.870548	64.320448	104.8313	47.469819	19.328411	9.094788637	23.82570486	21.79847263	31.70158702
CaWRKY35	24.412267	15.277804	17.247776	2.1554263	10.254178	3.141731051	2.001313464	0.160761343	0.328085982
CaWRKY36	27.933902	39.544505	23.546728	5.6497846	16.061483	10.77180142	13.18181738	4.929678561	17.50271864
CaWRKY37	15.673064	10.666407	14.82914	13.085637	16.159819	7.508038279	5.363520083	5.568685446	7.36463755
CaWRKY38	64.266254	69.316066	65.517943	63.262458	69.930058	23.09277321	22.6940339	33.5291118	30.78628328
CaWRKY39	3.9316989	17.006478	49.85377	0.8301618	4.3231358	0	0.256935094	0.257988264	0
CaWRKY40	0.2654692	0	8.8206248	0.3487724	1.933439	0	0	0	0
CaWRKY41	112.29558	161.62142	26.965895	8.2786813	21.483308	9.370250321	7.97357531	4.435121768	16.0455099
CaWRKY42	25.652633	308.24929	355.75319	20.44546	74.595856	0.482709865	1.300247293	0.261115394	0.532891171
CaWRKY43	0.5169289	0	0.0526326	0.0926099	0.0466716	0	1.160842022	0.690726093	0
CaWRKY44	21.752054	6.5326167	4.0405628	1.2546337	8.5358427	0.411879135	1.262004725	11.01469802	3.182879393
CaWRKY45	22.806729	9.9082686	109.66398	1.6474235	7.2805207	0.249612309	0.392213534	0.236292724	0.843906722
CaWRKY46	1.062185	0.1649293	0.183022	1.046621	4.2196371	0.079290321	0.149505786	0.22517791	0.153183002
CaWRKY47	0	0	0	0	0.1323247	0.064648643	0.060949092	0	0.249792736

CaWRKY48	16.95976	25.15042	8.5031165	5.1883307	7.9657409	0.831823788	1.960555644	1.968591907	6.083724924
CaWRKY49	1.3027653	0.4382842	4.1340943	0.5348651	0.5391007	0	0	0	0
CaWRKY50	5.6359976	21.331087	39.902725	3.7683883	43.679652	1.660332522	0.736620784	1.479280344	1.320792495
CaWRKY51	0	0	0.5002602	4.5662194	4.935082	7.260350418	7.815414977	1.846458858	0.314025154
CaWRKY52	0	0.2792608	0.1549478	0.7497577	1.9922873	5.034584559	5.569200794	1.207369853	0.194528857
CaWRKY53	64.427184	48.250542	47.071322	19.341418	19.589449	13.48694453	19.57520975	9.871597767	35.63641875
CaWRKY54	15.607805	18.8263	9.0387915	2.250601	5.973516	2.95536652	4.666959033	18.60447183	12.56100618
CaWRKY55	4.5355533	0.8765685	2.7020224	0.1901742	0.9584013	0	0	0	0.452299605
CaWRKY56	4.4903826	2.1401326	17.183138	0.8603361	3.22084	0	0	0	0
CaWRKY57	18.739778	42.581	5.4996549	1.0861875	1.0947892	0.583495441	1.283577456	0.184119829	3.381809355
CaWRKY58	0	0.3430051	0	0	0.3375239	0	0	0	0
CaWRKY59	62.258469	35.431821	7.9866095	1.0809904	12.938417	1.064623261	2.258324245	3.527348307	27.89498302
CaWRKY60	98.687425	55.915006	22.534377	6.4222588	15.268114	6.32502007	4.083405018	10.08765288	4.183839671
CaWRKY61	1.046865	24.935243	59.210479	0.6189153	4.4706852	0	0	0	5.299174481

Note: Fruit-Dev5, Mature green; The full size green fruit (5-6 cm); Fruit-Dev6, Breaker (fruit turning red); Fruit-Dev7, Breaker plus 3 days; Fruit-Dev8, Breaker plus 5 days

Name	Gene ID	Root	Leaf	Bud	Flower	Fruit-Dev5	Fruit-Dev6	Fruit-Dev7
SlWRKY01	solyc12g014610.1.1	41.66	11.43	10.63	9.95	17.83	14.42	3.67
SlWRKY02	solyc02g021680.2.1	11.73	0.03	1.21	1.79	0.66	10.44	8.17
SlWRKY03	solyc05g007110.2.1	7.54	0.02	0.00	0.00	0.00	0.00	0.00
SlWRKY04	solyc12g096350.1.1	2.17	4.61	1.82	2.28	21.32	10.63	3.24
SlWRKY05	solyc07g056280.2.1	2.13	2.15	2.21	2.82	1.31	0.79	0.63
SlWRKY06	solyc07g047960.2.1	7.14	2.00	4.91	4.81	17.48	6.98	6.95
SlWRKY07	solyc01g079360.2.1	5.79	13.98	11.16	14.25	5.22	3.41	0.54
SlWRKY08	solyc03g007640.1.1	0.00	0.00	0.00	0.00	0.00	0.00	0.00

SlWRKY09	solyc04g050210.1.1	0.00	0.00	0.00	0.00	0.00	0.00	0.00
SlWRKY10	solyc01g058540.2.1	0.05	0.18	0.16	0.09	0.50	0.00	0.07
SlWRKY11	solyc07g066220.2.1	56.49	19.57	23.86	46.40	51.65	43.28	50.39
SlWRKY12	solyc06g066370.2.1	55.78	69.95	6.41	30.79	73.48	54.15	95.18
SlWRKY13	solyc03g082750.1.1	0.00	0.00	0.08	0.00	0.00	0.00	0.00
SlWRKY14	solyc05g015850.2.1	13.92	0.79	1.19	1.51	15.64	2.89	1.41
SlWRKY15	solyc09g066010.1.1	19.90	2.57	2.87	2.53	2.42	1.23	1.15
SlWRKY16	solyc01g095630.2.1	17.32	43.41	9.53	9.63	50.43	19.40	44.89
SlWRKY17	solyc08g081610.2.1	3.55	0.76	0.63	0.58	0.16	0.00	0.00
SlWRKY18	solyc09g010960.2.1	1.00	0.33	0.26	0.00	0.00	0.00	0.00
SlWRKY19	solyc12g056750.1.1	2.89	0.00	0.00	0.00	0.00	0.00	0.00
SlWRKY20	solyc02g088340.2.1	17.77	83.52	10.34	21.24	45.44	228.13	34.35
SlWRKY21	solyc02g071130.2.1	4.92	0.32	0.04	0.68	1.00	0.22	0.32
SlWRKY22	solyc08g082110.2.1	9.35	9.40	0.49	1.14	9.02	2.71	1.14
SlWRKY23	solyc09g015770.2.1	9.84	23.99	12.95	11.71	73.82	39.28	124.04
SlWRKY24	solyc05g050040.1.1	0.00	0.00	0.00	0.00	0.00	0.00	0.00
SlWRKY25	solyc07g051840.2.1	13.85	6.46	8.93	13.57	5.71	12.48	27.19
SlWRKY26	solyc05g050340.2.1	3.01	2.76	1.42	2.17	5.54	5.36	2.00
SlWRKY27	solyc03g104810.2.1	36.91	30.27	63.43	117.49	32.62	36.00	40.13
SlWRKY28	solyc03g007380.1.1	3.62	0.51	0.04	0.43	0.88	0.31	0.11
SlWRKY29	solyc05g045800.1.1	0.00	0.00	0.00	0.00	0.00	0.00	0.00
SlWRKY30	solyc04g051690.2.1	3.35	1.40	0.24	1.58	2.07	1.10	0.32
SlWRKY31	solyc05g050050.1.1	0.00	0.00	0.00	0.00	0.00	0.00	0.00
SlWRKY32	solyc05g050300.1.1	0.00	0.00	0.00	0.00	0.00	0.00	0.00
SlWRKY33	solyc02g093050.2.1	23.15	11.22	11.68	6.40	8.31	2.78	4.61
SlWRKY34	solyc08g008280.2.1	7.55	6.53	0.62	1.85	40.29	15.96	11.23

SlWRKY35	solyc12g011200.1.1	4.12	1.55	2.32	1.10	0.00	0.00	0.00
SlWRKY35	solyc03g082810.1.1	0.00	0.00	2.42	0.00	0.00	0.00	0.00
SlWRKY37	solyc09g014990.2.1	12.62	9.57	0.17	1.23	17.51	2.41	1.30
SlWRKY38	solyc10g009550.2.1	1.21	3.55	1.23	0.67	11.30	3.99	4.37
SlWRKY38	solyc10g011910.2.1	9.84	2.66	0.48	0.42	3.59	0.83	0.54
SlWRKY39	solyc05g045710.1.1	0.00	0.00	0.00	0.00	0.00	0.00	0.00
SlWRKY40	solyc12g042590.1.1	0.00	0.23	0.00	0.31	0.17	0.00	0.00
SlWRKY41	solyc08g006320.2.1	100.24	90.02	18.55	31.37	79.44	43.06	52.97
SlWRKY42	solyc04g056360.2.1	0.07	0.04	25.48	13.29	0.00	0.00	0.00
SlWRKY43	solyc05g050060.1.1	0.00	0.00	0.00	0.00	0.00	0.00	0.00
SlWRKY44	solyc04g051540.2.1	2.23	1.32	0.31	0.24	0.00	0.00	0.00
SlWRKY45	solyc02g032950.2.1	2.65	2.08	1.82	2.73	0.75	0.06	0.05
SlWRKY46	solyc05g012770.2.1	21.33	3.27	9.38	6.26	2.80	7.17	12.09
SlWRKY48	solyc10g005680.1.1	0.00	0.00	0.04	0.00	0.00	0.00	0.00
SlWRKY49	solyc03g116890.2.1	6.14	2.62	2.20	1.13	21.83	10.66	23.27
SlWRKY50	solyc03g113120.2.1	3.63	0.09	0.00	0.00	0.00	0.00	0.00
SlWRKY51	solyc07g005650.2.1	6.27	3.01	4.49	9.17	7.36	9.28	8.80
SlWRKY52	solyc02g094270.1.1	1.42	0.00	0.00	0.00	0.61	0.00	0.10
SlWRKY53	solyc05g012500.2.1	9.33	4.18	5.95	7.66	28.41	37.28	40.56
SlWRKY54	solyc05g050330.2.1	6.12	4.84	4.09	5.42	2.78	3.88	3.39
SlWRKY55	solyc02g072190.2.1	29.27	2.04	0.42	0.24	0.51	0.46	0.72
SlWRKY56	solyc10g084380.1.1	0.24	8.16	10.91	5.67	0.18	0.00	0.00
SlWRKY57	solyc06g008610.2.1	26.77	6.14	8.99	17.02	22.12	7.57	19.36
SlWRKY58	solyc04g078550.2.1	62.06	18.93	11.73	30.60	52.76	30.65	53.14
SlWRKY59	solyc06g070990.2.1	10.56	0.17	0.00	0.08	0.63	0.03	0.00
SlWRKY60	solyc04g072070.2.1	2.00	0.32	0.19	0.49	1.50	0.00	0.13

SlWRKY61	solyc07g065260.2.1	40.78	14.72	15.39	12.43	24.02	22.06	12.38
SlWRKY62	solyc06g048870.1.1	0.42	0.07	0.00	0.00	0.00	0.00	0.00
SlWRKY63	solyc01g079260.2.1	15.86	3.45	5.34	3.83	13.08	12.32	75.66
SlWRKY64	solyc08g062490.2.1	6.30	4.62	1.63	1.31	1.84	0.31	0.45
SlWRKY65	solyc05g053380.2.1	0.67	0.23	0.28	0.10	0.20	0.13	0.26
SlWRKY66	solyc01g095100.2.1	12.40	1.87	1.76	2.92	1.26	0.88	0.24
SlWRKY67	solyc05g014040.1.1	0.00	0.00	0.00	0.00	0.00	0.00	0.00
SlWRKY68	solyc08g067360.2.1	1.36	0.16	0.00	0.06	0.41	0.00	0.26
SlWRKY69	solyc05g045880.1.1	0.00	0.00	0.00	0.00	0.00	0.00	0.00
SlWRKY70	solyc05g055750.2.1	0.11	0.03	100.91	3.92	0.00	0.05	0.00
SlWRKY71	solyc01g089960.2.1	0.22	0.76	2.05	1.56	0.00	0.09	0.00
SlWRKY72	solyc10g007970.1.1	1.80	0.00	0.06	0.00	0.40	0.12	0.00
SlWRKY73	solyc06g068460.2.1	12.75	68.70	8.66	10.31	86.79	82.67	49.98
SlWRKY74	solyc02g080890.2.1	6.92	1.84	1.44	3.67	4.33	0.61	5.62
SlWRKY75	solyc12g006170.1.1	21.25	8.65	14.18	24.68	14.45	18.31	18.26
SlWRKY76	solyc03g095770.2.1	15.44	22.14	10.52	2.75	74.87	14.89	24.66
SlWRKY77	solyc08g081630.1.1	0.30	0.21	0.00	0.00	0.00	0.00	0.00
SlWRKY78	solyc07g055280.2.1	38.11	4.22	4.05	7.95	1.57	2.30	0.79
SlWRKY79	solyc08g067340.2.1	8.15	0.65	0.29	1.17	14.67	4.05	22.47
SlWRKY80	solyc01g104550.2.1	2.35	1.07	0.99	1.10	0.00	0.00	0.00
SlWRKY81	solyc02g067430.2.1	7.98	0.00	0.00	0.00	0.00	0.00	0.00

Note. Fruit-Dev5, Mature green; Fruit-Dev6, Breaker (fruit turning red); Fruit-Dev7, Breaker plus 10 days

Supplemental Table S5. Average expression level of different groups of fruit-expressed CaWRKYs during fruit maturation.

Group Type	Fruit-Dev5	Fruit-Dev6	Fruit-Dev7	Fruit-Dev8
I	14.14	18.45	28.80	37.94
IIa	9.68	29.22	52.84	5.89
IIb	7.24	24.33	53.33	101.70
IIc	3.92	5.66	11.75	16.95
IId	14.19	11.74	20.63	9.58
IIe	4.42	5.19	4.92	8.63
III	0.08	1.88	1.65	17.65
NG	2.52	2.78	0.83	15.81
All CaWRKYs	8.66	11.85	20.12	26.13

Supplemental Table S6. Number of stress-regulated CaWRKYs in pepper during fruit maturation.

	High Salinity		Drought		Heat	
	+	-	+	-	+	-
Fruit-Dev5	15	2	1	6	11	3
Fruit-Dev6	17	2	2	6	13	4
Fruit-Dev7	16	0	1	5	11	4
Fruit-Dev8	20	2	2	9	17	3
All CaWRKYs	23	3	3	11	19	6

Supplemental Table S7. The responses of CaWRKYs to plant hormones and abiotic stresses

		CK	SA	JA	ABA	BR	Salt	Drought	Heat
CaWRKY01	Ave	1.000002163	0.0004272	0.0082695	0.0014499	0.1646833	0.0101448	0.004862833	0.0349321
	Std	0.112346456	0.0001321	0.0021345	0.0003245	0.052163	0.0015499	0.001608086	0.0230706
CaWRKY3	Ave	1.007201836	4.6375739	1.5977337	2.874034	4.6803781	4.5464655	0.965270777	6.9359173
	Std	0.144409877	4.1628885	0.1038787	0.2512212	0.2856751	0.2203404	0.091287701	1.2530069
CaWRKY6	Ave	1.010677973	8.4400879	0.9343689	1.9214835	7.7307793	3.8798271	0.318902215	14.638979
	Std	0.175976592	0.4878001	0.1040917	0.0170606	0.182163	0.3251896	0.10154988	0.8809928
CaWRKY8	Ave	1.009649294	5.7092762	0.3192433	0.4686057	1.9677172	3.2712521	0.25265012	0.5647711
	Std	0.175667531	0.2017363	0.0194499	0.0159017	0.1584799	0.2542858	0.072185066	0.0730706
CaWRKY10	Ave	1.16147884	17.650867	2.8429505	7.2735604	12.763186	42.088937	22.61953855	5.6023432
	Std	0.641608086	0.8714628	1.2526694	4.2195773	1.0793397	7.0008427	3.875232986	1.8972487
CaWRKY11	Ave	1.002873078	3.9054422	0.9645419	1.8398036	5.7437775	8.7784394	1.134970615	5.6150323
	Std	0.091489389	0.3125412	0.2591686	0.08366	0.7144468	1.1181348	0.557095826	1.2144499
CaWRKY12	Ave	1.004816613	0.3547992	0.0939694	0.1671429	0.3133667	0.9962894	0.042082052	3.1931725
	Std	0.121893593	0.0183834	0.0037585	0.0244385	0.0062908	0.1226654	0.010243133	0.2612925
CaWRKY14	Ave	1.021475614	13.266748	0.1766798	0.5434357	67.678526	18.276431	0.531535134	2.1622629
	Std	0.24548359	1.2848633	0.1353409	0.5893501	3.545675	0.9936148	0.206237071	0.6469807
CaWRKY20	Ave	1.807274719	17.61032	0	3.7567893	0.6870974	22.187488	9.108361518	53.224926
	Std	2.230157167	1.4120822	0	2.123442	0.165681	4.6648647	3.981347258	15.158353
CaWRKY21	Ave	1.00466105	1.0357431	1.6884771	1.1411684	1.3313411	1.8305407	0.387828555	1.0498021
	Std	0.118105188	0.0799693	0.0872731	0.1121974	0.1219446	0.2253671	0.12656385	0.1132647
CaWRKY22	Ave	1.016555713	2.8690227	1.4460656	1.1843925	2.3646752	4.6997355	0.840487161	0.2035604
	Std	0.214398688	0.1697935	0.1047777	0.1264647	0.1730422	0.241597	0.296865517	0.0607279
CaWRKY23	Ave	1.144724945	13.583813	7.6970842	3.0687397	5.685456	9.1936257	1.244721181	20.378946
	Std	0.605858591	0.4893473	1.9811931	1.0191985	0.8151275	3.2840979	0.31965637	4.7044807

CaWRKY24	Ave	1.008625556	0.6299722	1.1518029	0.8823696	0.9353522	1.0286166	0.272132703	0.7746531
	Std	0.160292448	0.0264335	0.0844304	0.0563444	0.0513152	0.1113181	0.080045444	0.1310406
CaWRKY27	Ave	1.012460496	1.1480897	2.7831596	1.6926889	1.5792536	1.9767796	0.147188531	2.0929202
	Std	0.200712426	0.0539634	0.3231346	0.1313841	0.1366592	0.0380019	0.050835067	0.0600027
CaWRKY28	Ave	1.005665726	2.237654	0.8126021	0.8150683	1.4935005	3.4901656	0.667657819	20.688429
	Std	0.12873275	0.0824345	0.03013	0.0384446	0.059461	0.2394018	0.213827528	1.8105664
CaWRKY30	Ave	1.01864804	2.0974413	3.1549716	3.6565171	1.589065	3.6527199	1.177173777	1.9282291
	Std	0.248469785	0.1673693	0.3362219	0.1123348	0.171946	0.4309715	0.405492144	0.4283116
CaWRKY31	Ave	1.10547778	2.9507315	1.3486098	1.2114224	2.4491254	1.4415459	0.375533882	0.5803925
	Std	0.591483041	0.1492595	0.1605235	0.1601744	0.2237192	0.1815454	0.141514321	0.0372929
CaWRKY33	Ave	1.020273769	1.4451136	2.8711204	1.984679	1.8973567	4.318741	0.758664742	1.7707125
	Std	0.236333809	0.0195077	0.2479759	0.1920511	0.1924784	0.3356441	0.232069751	0.1889597
CaWRKY34	Ave	1.033635203	2.1358143	1.5570387	1.3770478	4.5263045	3.0957396	0.405153764	4.8666209
	Std	0.326563896	0.0285005	0.1608609	0.051231	0.4505064	0.3462879	0.153327734	0.7604319
CaWRKY35	Ave	1.000000189	28.140877	0.0722466	0.0408772	78.505134	5.8154446	0.016141636	0.0097947
	Std	0.221752295	5.3776685	0.0266713	0.0185384	12.443125	1.337683	0.08760376	0.007862
CaWRKY36	Ave	1.006676503	1.8566108	1.1019527	3.5349264	1.3043095	14.838544	0.239487414	1.167124
	Std	0.139068431	0.3376824	0.0898959	0.9404318	0.3169967	1.4935798	0.127601671	0.1769144
CaWRKY37	Ave	1.013336131	0.9408327	6.8469759	4.0769668	0.5622729	5.1146812	0.802751551	0.2296615
	Std	0.194442295	0.0420685	0.5586713	0.8126384	0.0095246	0.7136407	0.292831581	0.1076038
CaWRKY38	Ave	1.008320727	0.8781589	4.845222	4.0772165	1.0791144	7.7018535	1.387228584	4.9845674
	Std	0.163157807	0.0335127	0.1161155	0.4422484	0.0402829	0.5362875	0.420770703	0.777862
CaWRKY41	Ave	1.002945149	0.1851042	0.4065329	0.2906576	0.1458645	0.3624985	0.063869722	3.2941168
	Std	0.09513602	0.0185489	0.0355514	0.0272589	0.0049255	0.0568202	0.036952872	0.4216427
CaWRKY44	Ave	1.045832858	3.5694737	0.4045236	0.6294742	0.597579	1.1358398	0.206027286	0.065728
	Std	0.400518584	0.5159653	0.0895193	0.2015095	0.2103341	0.2268544	0.03872342	0.0247197

CaWRKY48	Ave	1.004099641	0.78435	4.9252407	3.8545205	1.1871047	2.8993368	1.84123213	12.426523
	Std	0.110626742	0.0535732	0.4125952	0.2929023	0.2245636	0.3505157	0.746411739	1.3834557
CaWRKY51	Ave	1.325295921	1.2021673	24.0471	11.32895	0.0648392	14.029733	0.487919547	7.1612131
	Std	1.191760226	0.1021138	2.9282207	3.0746711	0.019518	5.6952499	0.162340595	6.5581791
CaWRKY52	Ave	0.999999912	0.1011448	0.0449383	0.041783	1.9887092	0.0323878	0.056785994	0.2273399
	Std	0.266500721	0.0364778	0.0111298	0.0154888	0.446195	0.0202797	0.027053331	0.0843395
CaWRKY53	Ave	1.731575343	3.1478597	291.46907	190.77484	3.7292766	595.09345	37.84736916	192.24884
	Std	1.923700721	0.1527778	10.23433	12.078149	0.557195	12.552671	14.27893908	35.366769
CaWRKY54	Ave	1.0005833	0.3736952	0.3394166	0.4084207	0.1713456	0.8302797	0.148975012	0.2705346
	Std	0.041917183	0.0402157	0.0206604	0.1051079	0.0328816	0.123862	0.083965606	0.0616695
CaWRKY57	Ave	1.276815387	1.4160823	5.173194	7.4126386	0.1486124	1.6744436	1.351139367	0.881399
	Std	0.867221263	0.1721788	4.0279858	1.2251828	0.0861721	1.043175	0.333962652	0.3880703
CaWRKY59	Ave	1.059390607	2.1090945	3.7050435	2.5721294	0.7662121	38.016948	1.777504773	6.2061238
	Std	0.423921545	0.1744235	0.6024794	0.1549152	0.1679743	1.9588988	0.746653922	0.3066114
CaWRKY60	Ave	1.018312937	0.7861316	0.309941	0.2105835	10.194487	0.3748784	0.456124206	0.3378427
	Std	0.245298437	0.0952438	0.0544031	0.1680706	0.8080042	0.1433448	0.267780321	0.2030848
CaWRKY61	Ave	1.069357728	1.9854401	3.7737648	71.922503	1.8891433	22.087198	1.810932487	24.185779
	Std	0.435716353	0.1476707	1.9903373	0.8944792	0.1885376	4.2368118	2.008429967	10.346528

Red:Up regulated; Green:Down regulated

Supplemental Table S8. Number of hormone-regulated CaWRKYs in pepper during fruit maturation

	SA		JA		ABA		BR	
	+	-	+	-	+	-	+	-
Fruit-Dev5	11	2	8	4	10	3	8	2
Fruit-Dev6	13	2	9	5	12	2	10	3
Fruit-Dev7	14	1	8	5	10	2	11	10
Fruit-Dev8	15	4	10	7	15	4	9	4
All CaWRKYs	19	5	13	12	17	6	13	5

Supplemental Table S9 Fruit-expressing CaWRKYS are regulated by ABA and other abiotic stresses

Name	Group	ABA regulation	Early mature	Late mature	Salt	Heat	Drought
CaWRKY01	NG	0.00±0.00	-	+	0.01±0.0	0.03±0.02	0.00±0.00
CaWRKY03	IIc	2.87±0.25	+	+	4.55±0.22	6.94±1.25	
CaWRKY06	III	7.73±0.18	-	+	3.88±0.33	14.64±0.88	0.32±0.10
CaWRKY08	IId	0.47±0.02	-	+	3.27±0.25		0.25±0.07
CaWRKY10	IIe	7.27±4.22	+	+	42.09±7.00	5.60±1.90	22.62±3.88
CaWRKY11	IIb	1.84±0.08	+	+	8.78±1.12	5.62±1.21	
CaWRKY12	NG	0.17±0.03	-	+		3.19±0.26	0.04±0.01
CaWRKY14	IIc		+	+	18.28±0.99		2.16±0.65
CaWRKY20	III	3.76±2.12	-	+	22.19±4.66	53.22±15.16	9.10±3.98
CaWRKY21	I		+	+	1.83±0.23	0.39±0.13	0.39±0.13
CaWRKY22	IId		+	+	4.70±0.24	0.20±0.06	
CaWRKY23	IId	3.07±1.02	+	+	9.19±3.28	20.38±4.70	
CaWRKY24	I		+	+		0.27±0.08	0.27±0.08
CaWRKY27	IIa	1.69±0.13	+	+	1.98±0.04	2.09±0.06	0.15±0.05
CaWRKY28	I		+	+	3.49±0.24	20.69±1.81	
CaWRKY30	IId	3.66±0.11	+	+	3.65±0.43		
CaWRKY31	I		+	+			
CaWRKY33	I	1.98±0.19	+	+	4.32±0.34	1.77±0.19	

CaWRKY34	Ⅱb		+	+	3.10±0.35	4.87±0.76	
CaWRKY36	Ⅱc	3.53±0.94	+	+	14.84±1.49		0.24±0.13
CaWRKY37	Ⅰ	4.08±0.81	+	+	5.11±0.71	0.23±0.11	
CaWRKY38	Ⅰ	4.08±0.44	+	+	7.70±0.54	4.98±0.78	
CaWRKY41	Ⅱe	0.29±0.03	+	+	0.36±0.06	3.29±0.42	0.06±0.04
CaWRKY44	Ⅱc		-	+		0.07±0.02	0.21±0.04
CaWRKY48	Ⅱe	3.85±0.29	-	+	2.90±0.35	12.43±1.38	
CaWRKY51	Ⅰ	11.3±3.07	+	-	14.03±5.70	7.16±6.56	
CaWRKY52	NG	0.42±0.01	+	-	0.03±0.02	0.23±0.08	0.06±0.03
CaWRKY53	Ⅰ	190.77±12.08	+	+	595.09±12.55	192.25±35.37	37.85±14.28
CaWRKY54	Ⅱc		-	+		0.27±0.06	
CaWRKY59	Ⅱc	2.57±0.15	-	+	38.02±1.96	6.21±0.31	
CaWRKY60	Ⅱd	0.21±0.17	+	+			
CaWRKY61	Ⅱc	71.92±0.89	-	+	22.09±4.24	24.19±10.35	

"+" and "-" represent "expressed" or "not expressed", respectively. Numbers of ABA, Salt, Heat and drought indicate the relative expressions of *CaWRKY* genes under corresponding treatment (The expression genes in control materials are set as 1.0).

Supplemental Table S10 Primers used for qRT-PCR in this study

Primer	Sequence (5'→ 3')	Size (bp)
CaWRKY01-F	ATGGAAGGTGGTGAACAA	255
CaWRKY01-R	CCCTGCATAGCCTAGTTC	
CaWRKY04-F	TAGGGTTTAGCCACAATG	298
CaWRKY04-R	TAACTCCTGGGATGAAGA	
CaWRKY13-F	TTGAGAAATCCGTTGATG	188
CaWRKY13-R	ATGAGAAGGAGAAGACCC	
CaWRKY16-F	CAGGGATGAAGAAGATGA	282
CaWRKY16-R	GTGGCTGAAAGAGGAAGT	
CaWRKY24-F	ATGAGTTTAATGGTGGCT	151
CaWRKY25-R	AAGACTGGGATAATGCTG	
CaWRKY26-F	TGCGAGAAGTAATGGAAG	191
CaWRKY26-R	TAGGCTAAGGGAAACAAG	
CaWRKY34-F	CGGAAGCAAGTTCAAAGA	264
CaWRKY34-R	TAGGTCCAATGTAACAGTAGG	
CaWRKY38-F	ACTCGCTTCAATCTCACC	290
CaWRKY38-R	AACTTGTTTCTGCCCGTA	
CaWRKY50-F	AAATTTGATCAAGGATGCCA	223
CaWRKY50-R	TTTCCACCGTCATATCCAAA	

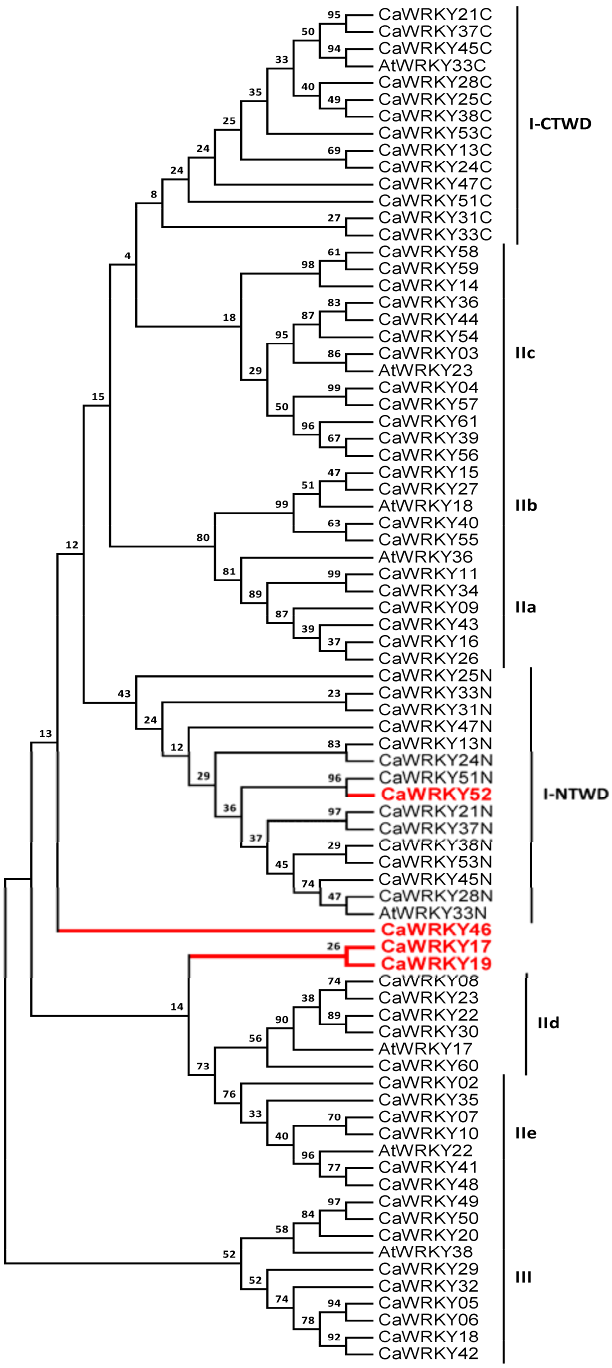

Supplemental Figure S1 Phylogenetic analysis of 61 CaWRKYs. The 74 WRKY domains (C- and N-terminal) derived from the 61 CaWRKY proteins were clustered using Clustal X 2.0, and loaded into MEGA 5.0 for phylogenetic tree construction. The clustered result is strictly consistent with the CaWRKY group classification. Four None-grouped (NG) members were identified according to this tree construction and labeled in red.
